# Prediction of microvascular invasion in hepatocellular carcinoma with conventional ultrasound, Sonazoid-enhanced ultrasound, and biochemical indicator: a multicenter study

**DOI:** 10.1186/s13244-024-01743-3

**Published:** 2024-10-28

**Authors:** Dan Lu, Li-Fan Wang, Hong Han, Lin-Lin Li, Wen-Tao Kong, Qian Zhou, Bo-Yang Zhou, Yi-Kang Sun, Hao-Hao Yin, Ming-Rui Zhu, Xin-Yuan Hu, Qing Lu, Han-Sheng Xia, Xi Wang, Chong-Ke Zhao, Jian-Hua Zhou, Hui-Xiong Xu

**Affiliations:** 1 Department of Ultrasound, Institute of Ultrasound in Medicine and Engineering, Zhongshan Hospital, Fudan University, Shanghai, China; 2 Shanghai Institute of Medical Imaging, Shanghai, China; 3 Department of Ultrasound, Sun Yat-sen University Cancer Center, State Key Laboratory of Oncology in South China, Guangdong, Provincial Clinical Research Center for Cancer, Guangzhou, China; 4 Department of Ultrasound, Nanjing DrumTower Hospital, The Affiliated Hospital of Nanjing University Medical School, Nanjing, China; 5 School of Medicine, Anhui University of Science and Technology, Anhui, China

**Keywords:** Hepatocellular carcinoma, Microvascular invasion, Contrast-enhanced ultrasound, Sonazoid, Kupffer-phase

## Abstract

**Purpose:**

To develop and validate a preoperative prediction model based on multimodal ultrasound and biochemical indicator for identifying microvascular invasion (MVI) in patients with a single hepatocellular carcinoma (HCC) ≤ 5 cm.

**Methods:**

From May 2022 to November 2023, a total of 318 patients with pathologically confirmed single HCC ≤ 5 cm from three institutions were enrolled. All of them underwent preoperative biochemical, conventional ultrasound (US), and contrast-enhanced ultrasound (CEUS) (Sonazoid, 0.6 mL, bolus injection) examinations. Univariate and multivariate logistic regression analyses on clinical information, biochemical indicator, and US imaging features were performed in the training set to seek independent predictors for MVI-positive. The models were constructed and evaluated using the area under the receiver operating characteristic curve (AUC), calibration curve, and decision curve analysis in both validation and test sets. Subgroup analyses in patients with different liver background and tumor sizes were conducted to further investigate the model’s performance.

**Results:**

Logistic regression analyses showed that obscure tumor boundary in B-mode US, intra-tumoral artery in pulsed-wave Doppler US, complete Kupffer-phase agent clearance in Sonazoid-CEUS, and biomedical indicator PIVKA-II were independently correlated with MVI-positive. The combined model comprising all predictors showed the highest AUC, which were 0.937 and 0.893 in the validation and test sets. Good calibration and prominent net benefit were achieved in both sets. No significant difference was found in subgroup analyses.

**Conclusions:**

The combination of biochemical indicator, conventional US, and Sonazoid-CEUS features could help preoperative MVI prediction in patients with a single HCC ≤ 5 cm.

**Critical relevance statement:**

Investigation of imaging features in conventional US, Sonazoid-CEUS, and biochemical indicators showed a significant relation with MVI-positivity in patients with a single HCC ≤ 5 cm, allowing the construction of a model for preoperative prediction of MVI status to help treatment decision making.

**Key Points:**

- MVI status is important for patients with a single HCC ≤ 5 cm.
- The model based on conventional US, Sonazoid-CEUS and PIVKA-II performs best for MVI prediction.
- The combined model has potential for preoperative prediction of MVI status.

**Graphical Abstract:**

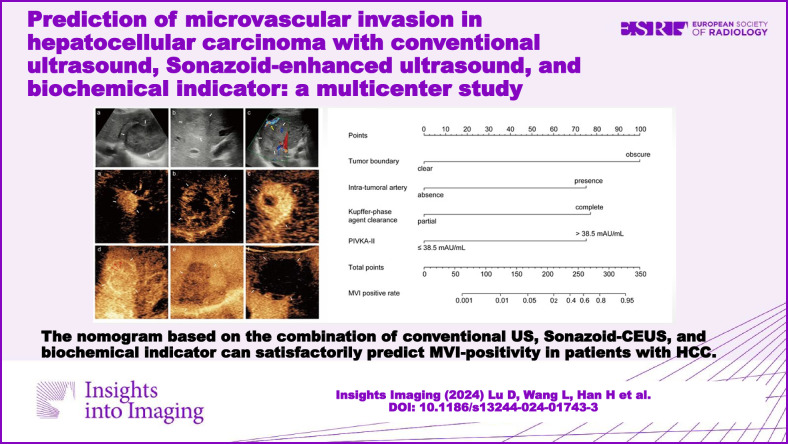

## Introduction

Hepatocellular carcinoma (HCC) is the sixth most common cancer and the third leading cause of cancer deaths with a relative 5-year survival rate of 18% [1]. Although surgery represents the main curative treatment option for patients, there is still a high incidence of tumor recurrence after liver resection, which occurs in up to 80% [2]. Microvascular invasion (MVI) is a histological feature defined as the presence of micro-metastasis in the vascular of normal liver tissue adjacent to the lesion. MVI has been reported to be an independent factor of postoperative tumor recurrence and poor clinical outcome [3, 4]. For patients with early and intermediate-stage HCC (e.g., a single tumor ≤ 5 cm), accurate preoperative identification of MVI status would provide clinical decision support for choosing a more appropriate curative therapy to eradicate potential MVI and improve long-term survival [5, 6].

Efforts have been devoted to exploring the relationship between preoperative parameters and MVI status. Some serum biomarkers have been reported to show great potential in the prediction of MVI status, such as alpha-fetoprotein (AFP) and vitamin K absence-II (PIVKA-II) [7, 8]. However, such biomarkers may also elevate in some benign liver diseases, and the cutoff value may vary in different test populations [9]. Except for serum biomarkers, some conventional ultrasound (US) imaging findings are considered to being promising in the preoperative prediction of MVI status. Some studies found that multinodular tumor morphology, tumor size, and obscure tumor boundary have been corroborated to have an association with MVI-positive [10–12].

Contrast-enhanced US (CEUS) is one of the most frequently employed imaging modalities for focal liver lesion in clinical practice, due to its non-radiation, real-time scanning, reproducibility, and convenience. As the second generation of microbubble US contrast agent, the post-vascular phase of Sonazoid has drawn great attention. Compared to pure blood pool agents, Sonazoid has a unique Kupffer-phase pattern with long-lasting and stable features [13]. Previous researches have shown that the Kupffer-phase pattern is helpful for the detection of obscure HCC on B-model US and early HCC [14, 15], as well as it has a correlation with the histological grade of HCC [16].

As far as we know, studies on the value of Sonazoid-CEUS on the prediction of MVI status are few and limited to a small number of cases or imaging examinations alone [17, 18]. Adding Sonazoid-CEUS to conventional US and biomedical indicators for the prediction of MVI status is well worth exploring. Therefore, the purpose of this study was to construct a preoperative prediction model based on conventional US, Sonazoid-CEUS, and biomedical indicators for preoperative prediction of MVI status in patients with a single HCC ≤ 5 cm.

## Method

This combined retrospective and prospective study was approved by the institutional Clinical Research Ethics Committee of Zhongshan Hospital, Fudan University (ID: B2022-569R), and the written informed consent from patients was waived. All procedures were performed according to the principles of the Declaration of Helsinki.

### Patients

From May 2022 to April 2023, 246 HCC patients who underwent conventional US and Sonazoid-enhanced CEUS examinations in Institution 1 (Zhongshan Hospital, Fudan University) were retrospectively enrolled as a model development dataset, following the inclusion criteria: (a) histologically confirmed HCC patients; (b) preoperative Sonazoid-enhanced CEUS examination within one week. The exclusion criteria were as follows: (a) benign lesions or non-HCC malignant lesions (*n* = 416); (b) HCC lesions with incomplete clinical or undefined pathological information (*n* = 5); (b) HCC lesions with history of prior adjuvant therapy before surgery (*n* = 21); (c) multiple HCC lesions (*n* = 43) or solitary HCC lesion with diameter > 5 cm (*n* = 23); (d) poor image quality influence the interpretation of lesion features (*n* = 6).

Following the same criteria used for the model development dataset, 72 HCC patients from Institution 2 (Sun Yat-sen University Cancer Center) and Institution 3 (Nanjing DrumTower Hospital) were prospectively enrolled as an independent external test set between May 2023 and November 2023.

**Fig. 1:**
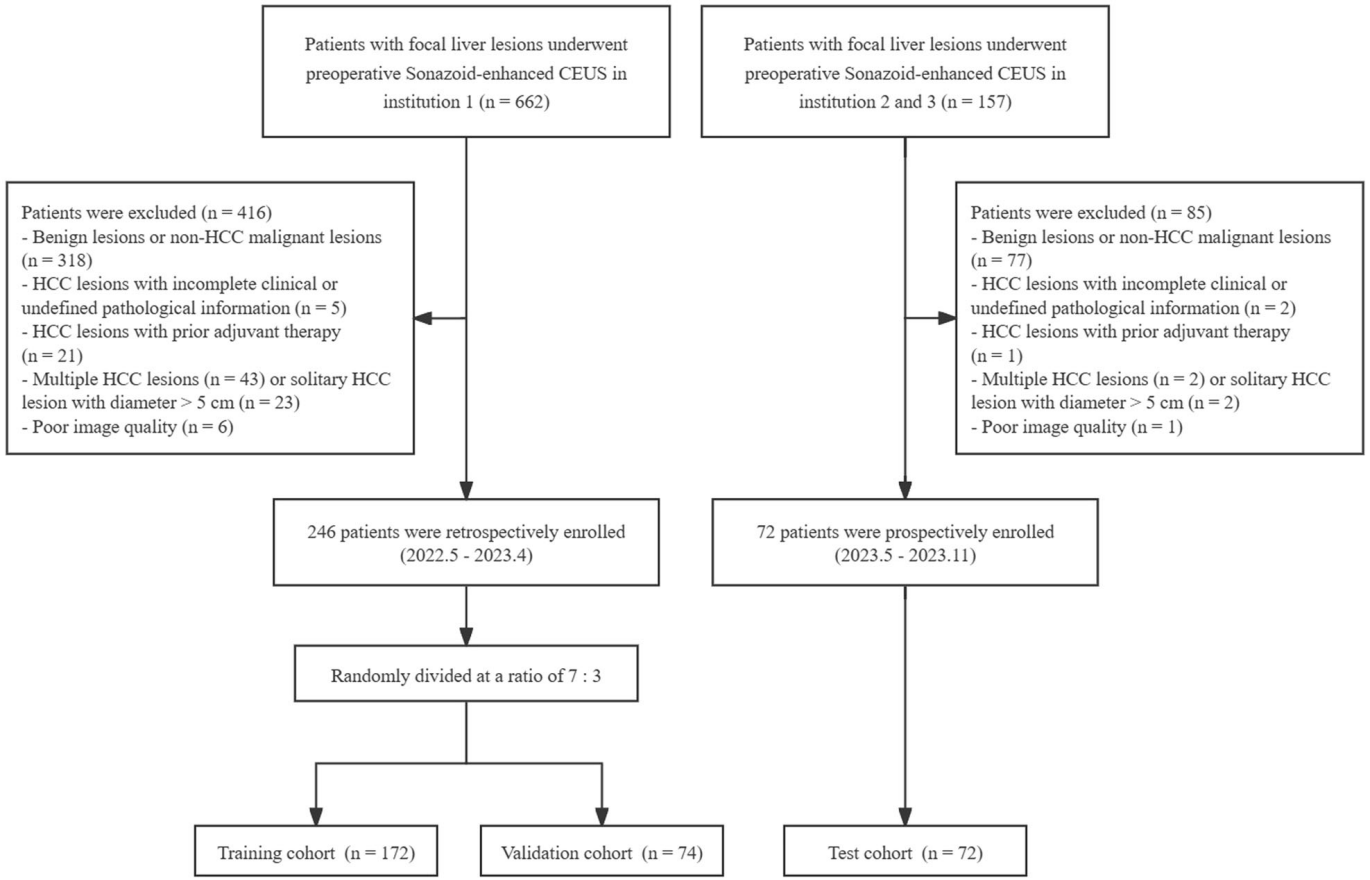
The participant flow chart for the present study

Patients were divided into MVI-positive group and MVI-negative group based on their postoperative pathology. A detailed flowchart of patient selection was presented in Fig. 1.

The clinical information and laboratory indices were acquired from the electronic medical record system, including sex, age, alcoholic factor, the infectious status of HBV and HCV, and serum levels of AFP and PIVKA-II.

### Histological assessment of MVI status

The surgical pathological examination included the evaluation of the MVI status of HCC lesions and liver cirrhosis status. The “7-point” sampling method was employed, that is, specimens were collected in a ratio of 1:1 in the 12, 3, 6, and 9 o’clock positions along the boundary between cancerous and adjacent non-neoplastic liver tissues. At least one tissue sample was collected from inside the tumor. One sample also was collected from the liver tissues in the non-neoplastic adjacent regions both ≤ 10 mm (proximal) and > 10 mm (distal) from the boundary of the tumor [19]. MVI refers to the presence of clusters of cancer cells in blood vessels with endothelial cell linings, detectable only under microscopy. All liver specimens were assessed by an experienced pathologist with more than 15 years of experience in liver pathology, who was unaware of the clinical information and preoperative multimodal US imaging findings.

### Conventional US and Sonozoid-CEUS examinations protocol

All the conventional US and Sonozoid-CEUS examinations were performed by one of six experienced radiologists with more than 10 years’ experience using Samsung RS80A (Samsung Ultrasound System, Seoul, Korea) equipped with a CA1-7A convex array transducer (frequency 1.0–7.0 MHz), Acuson Sequoia system (Siemens Medical Solutions, Mountain View, CA) equipped with a 5C1 convex array transducer (frequency 2.0–5.0 MHz), or LOGIQ E9 unit (GE Healthcare, Milwaukee, WI, United States) equipped with a C1-5-D convex array transducer (frequency 1.0–5.0 MHz).

B-mode and color Doppler US were performed before CEUS to identify the target lesion and assess their features. Real-time dynamic CEUS was performed under the mechanical index (0.18–0.20). B-mode and contrast-mode images were viewed on dual-screen displays and the scanner timer was used to record the time from contrast injection. Approximately 0.6 mL of Sonozoid contrast (GE Healthcare, Waukesha, WI, USA) was injected by a manual bolus injection for each patient followed by a 5 mL flush of 0.9% saline solution. The timer was started at the same time as the contrast injection. Based on the largest section of the tumor, dynamic scanning of the whole tumor was performed, and the dynamic images were stored for later analysis. The vascular phase images were acquired during the arterial phase (AP; 10–45 s after contrast injection), the portal venous phase (PVP; 45–120 s after contrast injection), and the delay phase (DP; 2–6 minutes after contrast injection). The post-vascular phases (Kupffer-phase, KP) images were obtained, starting at 6 min and lasting about 30 min. All multimodal US imaging data were exported to a hard disk for subsequent analysis.

### Evaluation of conventional US and Sonozoid-CEUS features

To ensure the reliability of the results, all the conventional US images and Sonozoid-CEUS videos were independently interpreted by two radiologists with more than 5 years of experience in liver CEUS. Both were blind to the clinical and pathological information except for the HCC diagnosis. If there was a disagreement between two readers, they consulted and decided on the final result. Interobserver agreement for qualitative features was analyzed by the Cohen kappa coefficient. The interclass correlation coefficient (ICC) was used to evaluate the interobserver agreement of quantitative features.

**Fig. 2:**
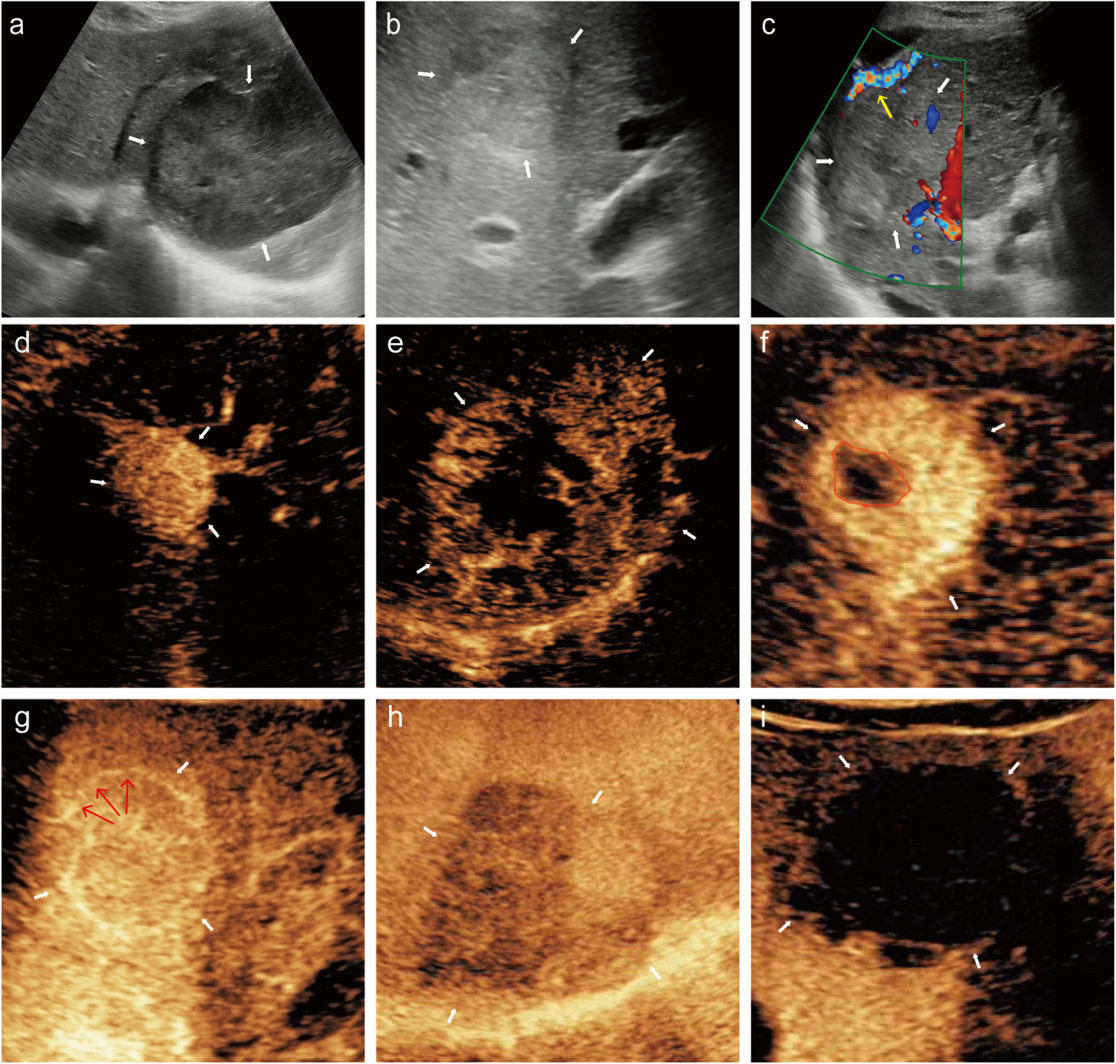
Illustrations of some conventional ultrasound and Sonazoid-CEUS features of HCC lesions (white arrows). **a** Clear boundary of the tumor. **b** Obscure boundary of tumor. **c** Intra-tumoral artery (yellow arrow). **d** Homogeneous AP enhancement pattern. **e** Heterogeneous AP enhancement pattern. **f** Tumor necrosis (non-enhancing area inside the tumor outlined with red lines). **g** Capsular enhancement in PVP or DP (outlined with red arrows). **h** Single nodular KP gross morphology with partial agent clearance. **i** Focal extra-nodular KP gross morphology with complete agent clearance. CEUS, contrast-enhanced ultrasound; AP, arterial phase; PVP, portal venous phase; DP, delayed phase; KP, Kupffer phase

The following conventional US features were collected: (a) largest tumor diameter; (b) tumor boundary, a clear border between the tumor and the liver parenchyma defined as clear tumor boundary, otherwise it is obscure tumor boundary; (c) intra-tumoral artery, defined as arterial blood flow was detected by pulsed Doppler US; Meanwhile, the following Sonozoid-CEUS features were also collected: (a) enhancement onset time, defined as contrast agent arrival time; (b) time to peak, defined as the time at which contrast concentration reached its maximum; (c) wash-out time, defined as the time at which the lesion began to show hypo-enhancement compared with the surrounding hepatic parenchyma; (d) AP enhancement pattern (homogeneous/heterogeneous); (e) tumor necrosis, defined as the presence of non-enhancing areas inside the tumor on AP images; (f) capsular enhancement, defined as peripheral rim of smooth hyper-enhancement on PVP or DP images; (g) KP gross morphology (single nodular type/focal extra-nodular type); (h) KP contrast agent clearance pattern, with almost no contrast agent retention in the tumor defined as complete clearance, otherwise, it is partial clearance (Fig. 2).

### Establishing and validation of the preoperative prediction model

Based on the univariable analysis of all the variables (including clinical, conventional US, and Sonozoid-CEUS features), variables with *p* values < 0.05 were included in the multivariable regression analysis for MVI prediction. A preoperative prediction model was established based on the result of multivariable regression analysis. In addition, a nomogram based on the independent predictors was built for the model visualization. The nomogram was generated by R (Version 4.2.3) software using the open source “rms” package.

### Subgroup analysis

In order to investigate the performance of the prediction model in HCC patients with different liver background (noncirrhosis or cirrhosis) and tumor sizes (< 3 cm or 3–5 cm), further subgroup analysis was conducted via the bootstrap resampling method (1000 times). 3 cm is one the cornerstone limits of HCC studies; most of the classification criteria or systems are based on this threshold (Barcelona Clinic Liver Cancer, Milan Criteria).

### Statistical analysis

The SPSS (Version 26.0) and R (Version 4.2.3) software were employed for data analyses. Normality was assessed using the Kolmogorov–Smirnov test. Continuous variables were described as the mean ± standard deviation. Differences in continuous variables were analyzed using the *t* test. The Chi-squared test was used to compare categorical variables. Receiver operating characteristic curve analysis was used to calculate the optimal cutoff values that were determined by maximizing the Youden index (i.e., sensitivity + specificity − 1). DeLong’s test was performed to compare the area under the curves (AUCs). Calibration ability was determined using the Hosmer-Lemeshow goodness-of-fit test and calibration curve analysis. Decision curve analysis was conducted by quantifying the net benefits at different threshold probabilities, which was generated by R software using “ggscidca” package. Differences in statistical significance were considered when *p* < 0.05.

## Result

### Clinical and multimodal US imaging characteristics

Eventually, there were 172, 74, and 72 patients in the training, validation, and test cohorts, respectively. The baseline characteristics were summarized in Table 1. A total number of 318 qualified patients with an average age of 57.90 ± 11.12 years (range, 27–83), including 250 males and 68 females were enrolled in this study. MVI-positive was identified in 26.2% (45/172), 23.0% (17/74), and 8.1% (13/72) patients in the training, validation, and test cohorts respectively, with no significant difference between the three cohorts (all *p* > 0.05). Additionally, MVI-positive was identified in 16.8% (22/131) patients with HCC < 3 cm and 28.3% (53/187) patients with HCC between 3 and 5 cm, respectively.

**Table 1:** Baseline characteristics of HCC patients with a single HCC ≤ 5 cm

Characteristic	Total (*n* = 318)	Training (*n* = 172)	Validation (*n* = 74)	Test (*n* = 72)
**Age, mean (SD), y**	57.90 ± 11.12	59.65 ± 10.98	57.12 ± 10.39	54.5 ± 11.4
**Gender**				
** Male**	250 (78.6)	130 (75.6)	57 (77.0)	63 (87.5)
** Female**	68 (21.4)	42 (24.4)	17 (23.0)	9 (12.5)
**HBsAg**				
** Negative**	59 (18.6)	39 (22.7)	15 (20.3)	5 (6.9)
** Positive**	259 (81.4)	133 (77.3)	59 (79.7)	67 (93.1)
**HCV-Ab**				
** Negative**	294 (92.45)	157 (91.28)	69 (93.24)	68 (94.44)
** Positive**	24 (7.55)	15 (8.72)	5 (6.76)	4 (5.56)
**Alcohol factor**				
** Absence**	187 (58.81)	103 (59.88)	34 (59.46)	40 (55.56)
** Presence**	131 (41.19)	69 (40.12)	30 (40.54)	32 (44.44)
**Liver background**				
** Non-cirrhosis**	160 (50.3)	86 (50.0)	32 (43.2)	21 (29.2)
** Cirrhosis**	158 (49.7)	86 (50.0)	42 (56.8)	51 (70.8)
**MVI**				
** Absence**	243 (76.4)	127 (73.8)	57 (77.0)	59 (81.9)
** Presence**	75 (23.6)	45 (26.2)	17 (23.0)	13 (8.1)
**AFP, ng/mL**	376.12 ± 1125.11	316.72 ± 722.47	273.69 ± 841.95	623.3 ± 1893.3
**PIVKA-II, mAU/mL**	371.57 ± 1099.43	416.58 ± 1317.99	236.41 ± 498.16	402.9 ± 965.9
**Tumor boundary**				
** Clear**	113 (35.5)	64 (37.2)	25 (33.8)	24 (33.3)
** Obscure**	205 (64.5)	108 (62.8)	49 (66.2)	48 (66.7)
**Tumor diameter, cm**	2.99 ± 1.21	3.07 ± 1.31	2.71 ± 1.00	3.10 ± 1.13
**Intra-tumoral artery**				
** Absence**	196 (61.6)	106 (61.6)	54 (73.0)	36 (50.0)
** Presence**	122 (38.4)	66 (38.4)	20 (27.0)	36 (50.0)
**Enhancement onset time, s**	19.26 ± 5.83	19.80 ± 5.33	21.22 ± 6.17	14.64 ± 2.82
** Time to Peak, s**	28.35 ± 8.76	30.12 ± 10.30	29.78 ± 7.97	21.82 ± 4.44
** Wash-out Time, s**	58.22 ± 18.58	62.10 ± 18.18	57.85 ± 22.41	58.15 ± 18.82
**AP enhancement pattern**				
** Homogeneous**	204 (64.2)	113 (65.7)	50 (67.6)	41 (56.9)
** Heterogeneous**	114 (35.8)	59 (34.3)	24 (32.4)	31 (43.1)
**Necrosis**				
** Absence**	280 (88.1)	155 (90.1)	65 (87.8)	60 (83.3)
** Presence**	38 (11.9)	22 (9.9)	9 (12.2)	12 (16.7)
**Capsular enhancement**				
** Absence**	256 (80.5)	141 (82.0)	59 (79.7)	56 (77.8)
** Presence**	62 (19.5)	31 (18.0)	15 (20.3)	16 (22.2)
**KP gross morphology**				
** Single nodular type**	273 (85.8)	142 (82.6)	63 (85.1)	68 (94.4)
** Focal extra-nodular type**	45 (14.2)	30 (17.4)	11 (14.9)	4 (5.6)
**KP agent clearance**				
** Completely clear**	104 (32.7)	64 (37.2)	23 (31.1)	17 (23.6)
** Partially clear**	214 (67.3)	108 (62.8)	51 (68.9)	55 (76.4)

Continuous variables are expressed in mean ± standard deviation. Categorical variables are expressed in counts and data in parentheses are percentages.

*HBsAg* hepatitis B surface antigen, *HCV-Ab* hepatitis C virus antibody, *AFP* alpha-fetoprotein, *AP* arterial phase, *KP* Kupffer phase

The interobserver agreement between qualitative US imaging features is listed in Table S1, and the agreement between different observers was more than 0.85. For quantitative multimodal US imaging features, ICCs > 0.90 were considered excellent (Table S2).

### Preoperative predictors for presence of MVI based on univariate and multivariate analysis

The results of the univariate and multivariate analysis of the clinical and multimodal US imaging characteristics in the training cohort are listed in Table 2.

**Table 2:** Influencing clinical and multimodal US imaging characteristics of microvascular invasion in the training cohort

Characteristic	Univariate analysis		Multivariate analysis	
	OR (95% CI)	*p* value	OR (95% CI)	*p* value
**Age, y**	1.02 (0.99, 1.05)	0.261	NA	/
**Gender**				
** Male vs female**	0.85 (0.39, 1.85)	0.854	NA	/
**HBsAg**				
** Positive vs negative**	0.47 (0.22, 1.00)	0.079	NA	/
**HCV-Ab**				
** Positive vs negative**	1.029 (0.301, 3.441)	0.963	NA	/
**Alcohol factor**				
** Presence vs absence**	1.125 (0.564, 2.564)	0.737	NA	/
**History of HCC primary vs recurrent**				
** Recurrent vs primary**	0.55 (0.22, 1.35)	0.178	NA	/
**Liver background non-cirrhosis vs cirrhosis**				
** Cirrhosis vs non-cirrhosis**	1.35 (0.68, 2.68)	0.404	NA	/
**AFP, ng/mL**			NA	/
** > 529 vs ≤ 529**	2.80 (1.19, 6.58)	0.018	3.12 (0.52, 18.29)	0.214
**PIVKA-II, mAU/mL**				
** > 38.5 vs ≤ 38.5**	11.40 (3.36, 38.70)	< 0.001	11.86 (2.07, 67.98)	0.005
**Tumor boundary smooth vs nonsmooth**				
** Obscure vs clear**	20.51 (4.76, 88.29)	< 0.001	38.53 (4.93, 301.21)	0.001
** Tumor diameter, cm**	1.36 (1.04, 1.78)	0.027	1.33 (0.72, 2.45)	0.357
**Intra-tumoral artery**				
** Presence vs absence**	7.83 (3.63, 16.93)	< 0.001	15.12 (4.02, 56.88)	< 0.001
** Enhancement onset time, s**	0.96 (0.90, 1.03)	0.269	NA	/
** Time to Peak, s**	0.98 (0.94, 1.02)	0.348	NA	/
** Wash-out Time, s**	1.00 (0.98, 1.02)	0.784	NA	/
**AP enhancement pattern**				
** Homogeneous vs heterogeneous**	0.99 (0.48, 2.01)	0.978	NA	/
**Necrosis**				
** Presence vs absence**	3.72 (1.34, 10.34)	0.006	0.84 (0.11, 6.50)	0.888
**Capsular enhancement**				
** Presence vs absence**	0.79 (0.32, 1.98)	0.581	NA	/
**KP gross morphology**				
** Focal extra-nodular vs singular**	4.97 (2.32, 10.66)	< 0.001	1.41 (0.42, 4.77)	0.511
**KP agent clearance**				
** Complete vs partial**	14.14 (6.09, 32.84)	< 0.001	22.81 (5.50, 94.66)	< 0.001

*HBsAg* hepatitis B surface antigen, *HCV-Ab* hepatitis C virus antibody, *AFP* alpha-fetoprotein, *OR* odds ratio, *AP* arterial phase, *KP* Kupffer phase, *NA* not applicable

Univariate analysis showed that the serum AFP and PIVKA-II level, tumor size, tumor boundary, intra-tumoral artery, necrosis, KP gross morphology, and KP agent clearance pattern were significantly related to MVI-positive (all *p* < 0.05).

On multivariate analysis, the serum PIVKA-II level, tumor boundary, intra-tumoral artery, and KP agent clearance pattern were independently associated with the presence of MVI (all *p* < 0.05). The OR values of obscure tumor boundary, presence of intra-tumoral artery, and complete KP agent clearance pattern were all > 1, which indicated that these US imaging features were positively correlated with the occurrence of MVI.

### Prediction model construction and validation

Three prediction models for MVI in HCC patients were developed based on the result of multivariate analysis. (a) The BD-US model, included two predictors acquired from B-mode and Doppler US: tumor boundary and intra-tumoral artery. (b) The BDS-US model, added the predictor of KP agent clearance observed on Sonazoid-CEUS into the BD-US model. (c) The combined model, combined all above US imaging features and the serum PIVKA-II.

**Fig. 3:**
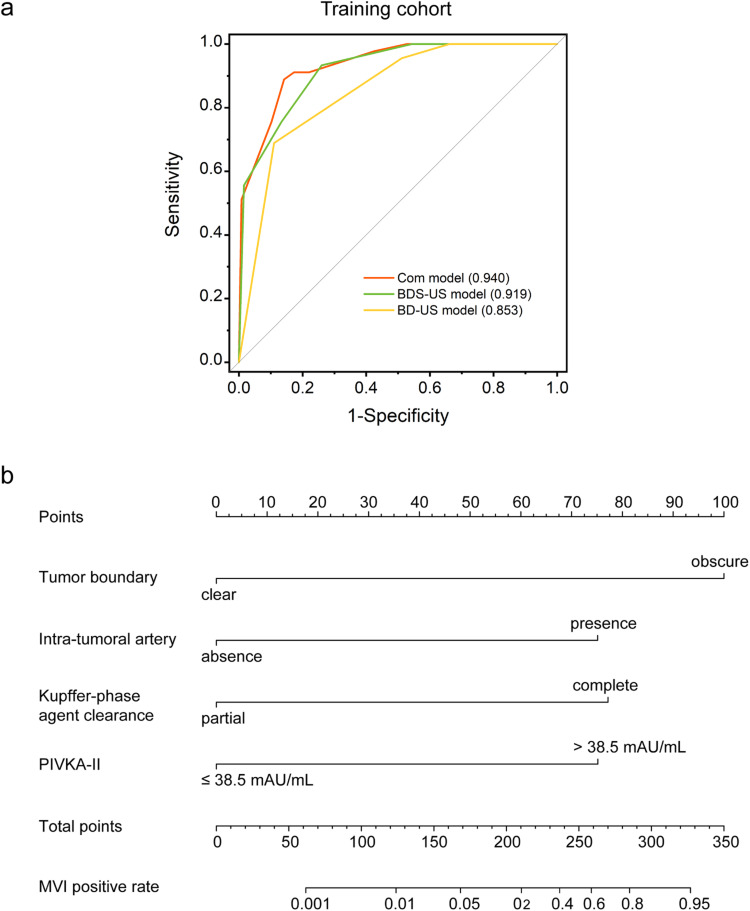
**a** Receiver operating characteristic (ROC) curves of BD-US model, BDS-US model and combined model in the training cohort. **b** Nomogram for predicting the probability of MVI in HCC patients. MVI, microvascular invasion. (BD-US model, model based on the features of tumor boundary and intra-tumoral artery in B-mode and Doppler US. BDS-US model, model based on the features of tumor boundary, intra-tumoral artery and Kupffer-phase agent clearance pattern in conventional US and Sonazoid CEUS. Combined model, model based on the combination of US features and serum PIVKA-II level)

**Fig. 4:**
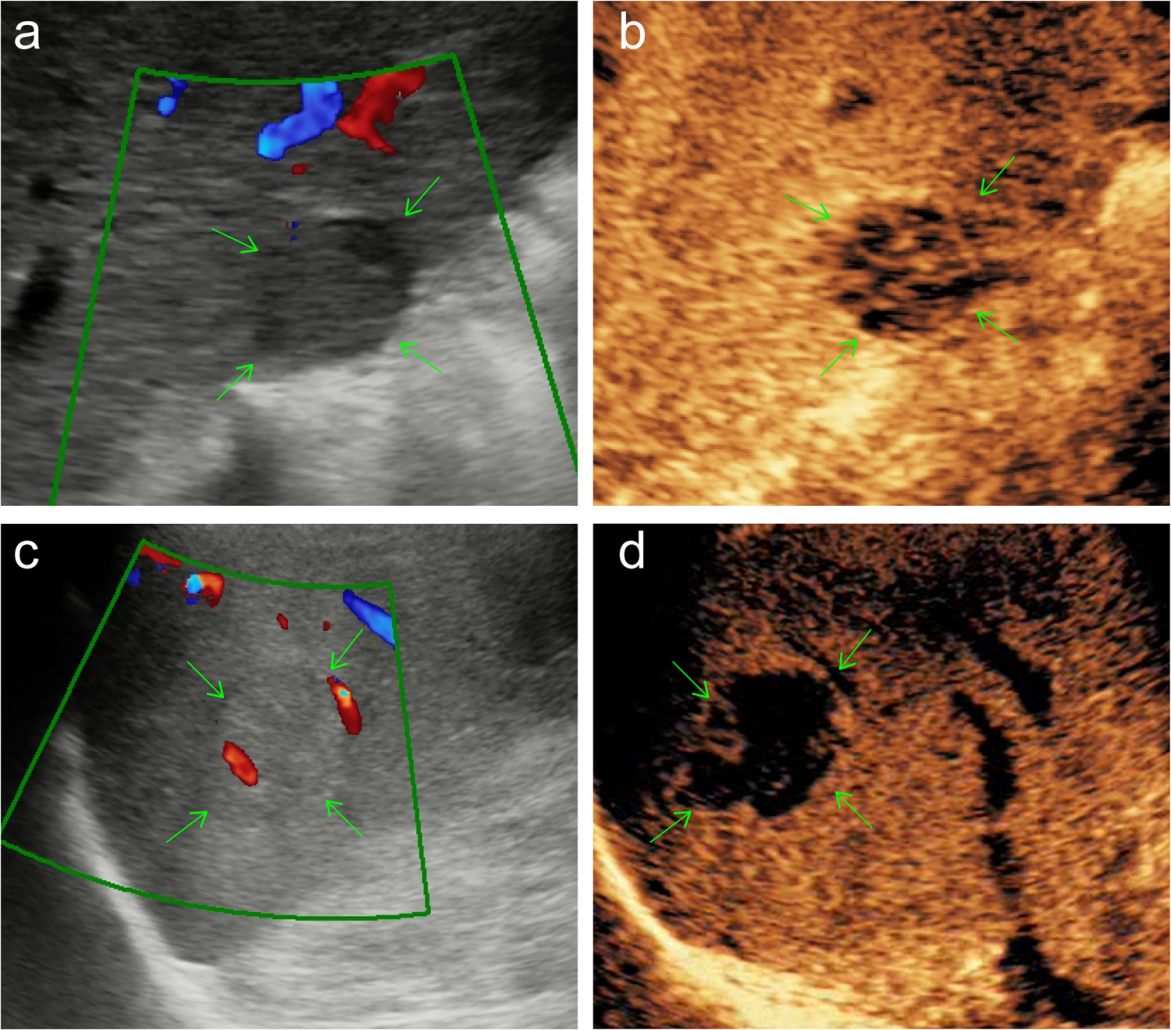
Examples correctly diagnosed by combined model. A case of an MVI-negative 46-year-old man with clear tumor boundary (**a**) and partial Kupffer phase agent clearance (**b**). A case of an MVI-positive 77-year-old man with obscure tumor boundary and intra-tumoral artery (**c**) on conventional US and complete Kupffer phase agent clearance on CEUS (**d**)

The AUCs of BD-US model, BDS-US model, and combined model were 0.853, 0.919, and 0.940 in the training cohort, respectively (Fig. 3a and Table 3). The predictive performance of the combined model was better than the BD-US model (*p* < 0.001) in the training cohort. A nomogram was drawn for the graphical representation of the predictive outcome of the combined model (Fig. 3b). In the validation cohort, the combined model showed a better performance with an AUC of 0.937 compared with the BD-US model (*p* = 0.003) and the BDS-US model (*p* = 0.031) (Table 3). In the test cohort, the combined model maintained a better performance with an AUC of 0.893 compared with the BD-US model and the BDS-US model (both *p* < 0.05). Examples correctly diagnosed by the combined model are shown in Fig. 4.

**Table 3:** Performance of the BD-US model, the BDS-US model, and the combined model

Models	Data sets	Sensitivity	Specificity	PPV	NPV	Accuracy	AUROC	*p* value
BD-US	Training cohort	0.689 (0.554, 0.824)	0.890 (0.835, 0.944)	0.689 (0.554, 0.824)	0.890 (0.835, 0.944)	0.837 (0.836, 0.839)	0.853 (0.763, 0.880)	< 0.001 (vs Com)
	Validation cohort	0.471 (0.233, 0.708)	0.912 (0.839, 0.986)	0.615 (0.351, 0.880)	0.852 (0.763, 0.941)	0.811 (0.807, 0.815)	0.763 (0.648, 0.878)	0.003 (vs Com)
	Test corhort	0.696 (0.548, 0.843)	0.780 (0.674, 0.885)	0.350 (0.141, 0.559)	0.885 (0.798, 0.971)	0.736 (0.731, 0.741)	0.696 (0.548, 0.843)	0.010 (vs Com)
BDS-US	Training cohort	0.933 (0.860, 1.000)	0.740 (0.664, 0.816)	0.560 (0.448, 0.672)	0.969 (0.935, 1.004)	0.791 (0.789, 0.793)	0.919 (0.878, 0.960)	0.225 (vs Com)
	Validation cohort	0.941 (0.829, 1.000)	0.842 (0.747, 0.937)	0.640 (0.452, 0.828)	0.980 (0.940, 1.019)	0.865 (0.862, 0.868)	0.899 (0.830, 0.969)	0.031 (vs Com)
	Test cohort	1.000 (1.000, 1.000)	0.661 (0.540, 0.782)	0.394 (0.227, 0.561)	1.000 (1.000, 1.000)	0.722 (0.717, 0.728)	0.816 (0.723, 0.909)	0.034 (vs Com)
combined	Training cohort	0.889 (0.797, 0.981)	0.858 (0.798, 0.919)	0.690 (0.571, 0.809)	0.956 (0.919, 0.994)	0.866 (0.865, 0.868)	0.940 (0.904, 0.975)	/
	Validation cohort	0.941 (0.829,1.000)	0.930 (0.864, 0.996)	0.800 (0.625, 0.975)	0.981 (0.946, 1.017)	0.932 (0.931, 0.934)	0.937 (0.880, 0.993)	/
	Test cohort	1.000 (1.000, 1.000)	0.797 (0.694, 0.899)	0.520 (0.324, 0.716)	1.000 (1.000, 1.000)	0.833 (0.830, 0.837)	0.893 (0.821, 0.966)	

*PPV* positive predictive value, *NPV* negative predictive value, *AUROC* area under the receiver operating characteristic curve. BD-US model, model based on the features of tumor boundary and intra-tumoral artery in conventional B-mode and Doppler US. BDS-US model, model based on the features of tumor boundary, intra-tumoral artery and Kupffer-phase agent clearance in conventional US and Sonazoid-CEUS. Combined model, model based on the combination of US features and serum PIVKA-II level

**Fig. 5:**
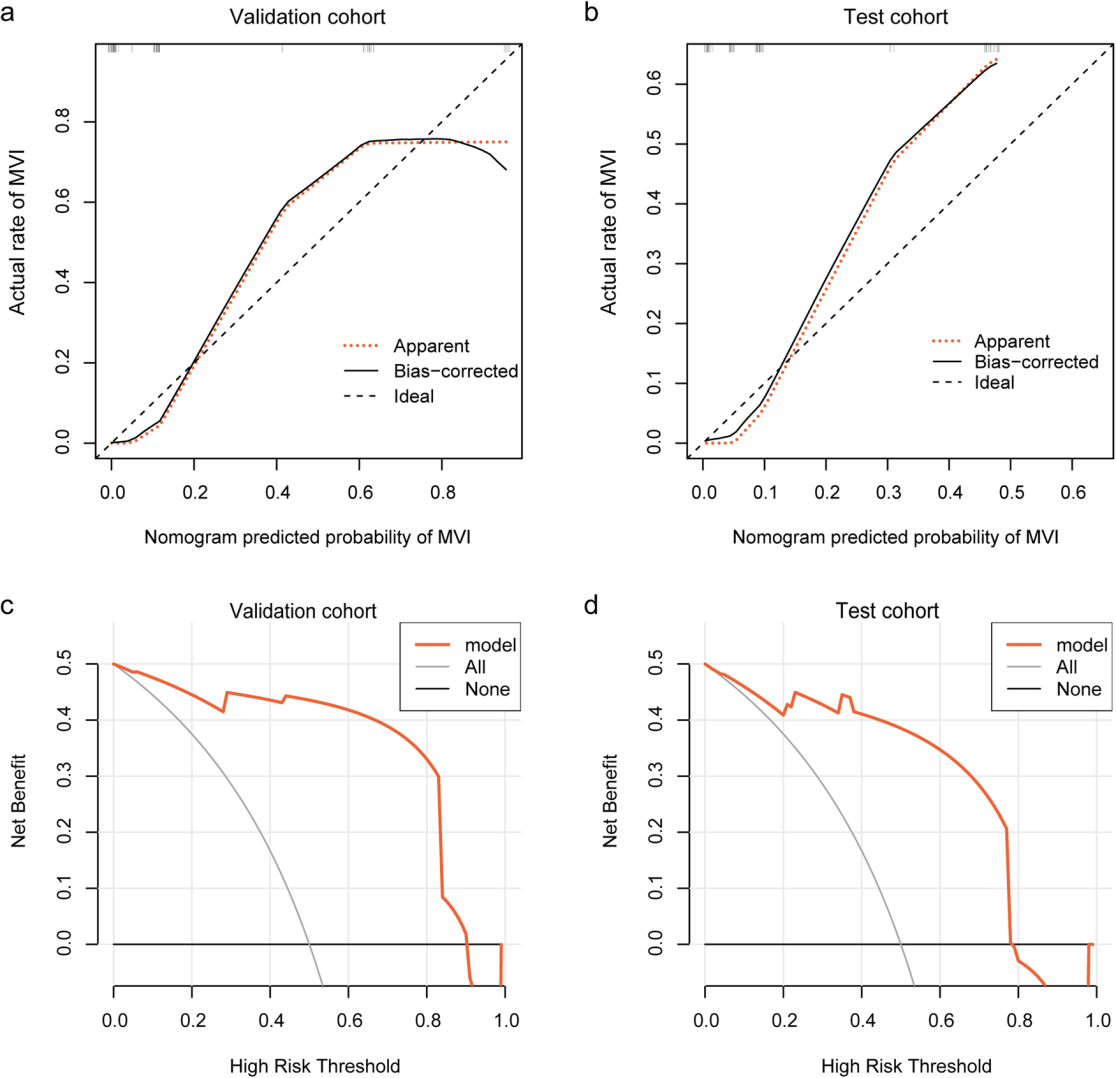
Calibration curves of the prediction model in the validation (**a**) and test cohorts (**b**). Decision curves for the prediction model in the validation (**c**) and test cohorts (**d**). MVI, microvascular invasion

The combined model was validated using the bootstrap validation method. Good calibration was observed both in the validation (Fig. 5a) and test (Fig. 5b) cohorts, with C-indexes of 0.937 and 0.893, respectively. The Hosmer-Lemeshow test yielded no significant difference between the predictive calibration curve and the ideal curve for MVI prediction both in the validation and test cohorts (*p* = 0.670 and 0.502, respectively). Decision curves for the prediction model were presented in Fig. 5c and d. In the validation cohort, the benefit value of the combined model was always higher than that for assuming all patients were MVI-positive (Fig. 5c). In the test cohort, the threshold probability of the decision curve was 3% and the corresponding net benefit was 0.486 (Fig. 5d). Decision curve analysis showed that employment of the model for preoperative MVI prediction was much more beneficial.

### Subgroup analysis

**Fig. 6:**
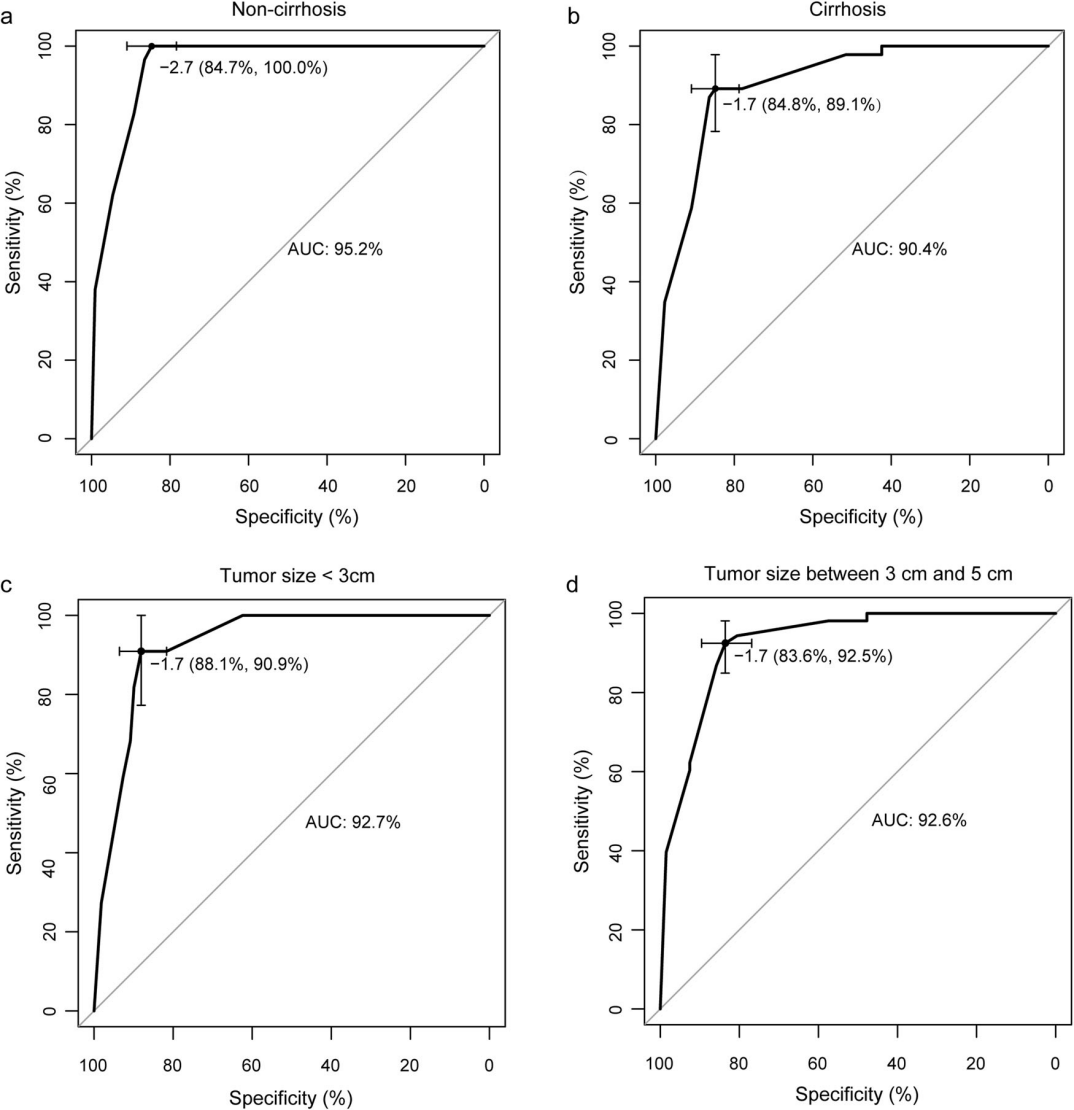
The MVI prediction performance of combined model in subgroups. ROC curves of for the combined model in patient with non-cirrhosis liver background (**a**), cirrhosis liver background (**b**), tumor diameter < 3 cm (**c**), and tumor diameter between 3 and 5 cm (**d**). MVI, microvascular invasion

In the cohort of non-cirrhosis (*n* = 140), the AUC, sensitivity, and specificity of the combined model were 0.952, 1.000, and 0.847, respectively (Fig. 6a). In the cohort of cirrhosis (*n* = 178), the AUC, sensitivity, and specificity of the combined model was 0.904, 0.891, and 0.848, respectively (Fig. 6b). In the cohort of tumor diameter < 3 cm (*n* = 131), the AUC, sensitivity and specificity of the combined model was 0.927, 0.909, and 0.881, respectively (Fig. 6c). In the cohort of tumor diameter between 3 and 5 cm (*n* = 187), the AUC, sensitivity, and specificity of the combined model was 0.926, 0.925, and 0.836, respectively (Fig. 6d). No significant differences was found in AUCs in patients with different liver background (*p* = 0.089) and tumor sizes (*p* = 0.984).

## Discussion

In this study, we found that serum level of PIVKA-II, obscure tumor boundary in B-mode US, intra-tumoral artery in color Doppler US, and complete agent clearance at KP in CEUS were independent factors of MVI in HCC patients with a single HCC ≤ 5 cm. Furthermore, we developed and validated a prediction model based on the incorporation of clinic-ultrasonic characteristics by means of a multicenter study. The combined model exhibited a higher AUC than the BD-US and BDS-US models (0.937 vs. 0.899 and 0.763, both *p* < 0.05) in the validation cohort. In the test cohort, the combined model still maintained a better performance with an AUC of 0.893 compared to the BD-US and BDS-US models (both *p* < 0.05). Good calibration and prominent net benefit were achieved in both training and validation cohorts. In subgroup analyses for patients with different liver background and tumor sizes, the AUCs of the combined model were all higher than 0.90, and no significant differences was found in the different groups. Therefore, our proposal prediction model was a non-invasive, easy-to-understand, and robust approach to determine the risk of MVI for HCC patients with a single HCC ≤ 5 cm.

To preoperatively identify HCC patients with high-risk recurrence, great efforts have been devoted to exploring reliable and convenient approaches for MVI prediction. Serum tumor markers, as economical and highly specific means, are routinely applied for tumor screening and monitoring. PIVKA-II was the only independent factor of MVI in the serum markers in this study. The serum half-life of PIVKA-II is much shorter than that of AFP which allows better sensitivity for the diagnosis of HCC and better performance in disease surveillance [20, 21]. Elevated serum PIVKA-II level has been proved to be closely associated with the loss of tumor suppressor p53, which accelerates the growth of cancer cells [22, 23]. The cutoff value of PIVKA-II for MVI prediction was 38.5 mAU/mL with an extremely high sensitivity (94.0–100.0%). However, it should not be neglected that PIVKA-II > 38.5 mAU/mL can also be detected in many MVI-negative patients. The poor specificity (36.2–53.5%) needed to be improved in conjunction with other indicators.

As for the conventional US imaging features, obscure tumor boundary and intra-tumoral artery were independent factors of MVI-positive. The imaging feature of obscure tumor boundary can be observed by most routine imaging modalities and has been identified as a potential predictor of MVI in many previous studies [24–26]. However, no matter in this work or in previous studies, the sensitivity of obscure tumor boundary only was high while the specificity was less than desirable (40.1–57.5%). Another conventional US feature of the intratumoral artery showed a moderate sensitivity (60.4–86.3%) and specificity (66.4–81.6%) in our study. The presence of an intratumoral artery means the tumor has built up its own blood supply system-known as angiogenesis [27]. It has been reported to be strongly correlated with macrotrabecular-massive hepatocellular carcinoma, which is a pathological subtype with poor prognosis [28]. Tumor angiogenesis accelerates the growth of tumor, thus the presence of more aggressive tumor behavior like MVI can be explained.

As for the Sonazoid-CEUS features, complete agent clearance at KP was an independent factor of MVI-positive. Many previous studies have explored the application of the blood-pool contrast agent Sonovue for MVI prediction [29]. Different from Sonovue, Sonazoid could be engulfed by Kupffer cells and enable KP imaging. As a kind of tissue-resident macrophages in liver, Kupffer cells are capable of eliminating foreign antigens, antigen-antibody complexes and cellular debris from the blood by phagocytic function [30]. Macrophages in HCCs are extremely heterogeneous with normal liver tissue. As disease progresses, cancer cells avidly deplete glucose, as a result lactate expression is extremely high in tumor microenvironment [31]. Lactate and tumor-associated cytokines favor the macrophage polarization toward an immunosuppressive M2-like state, which exhibit limited phagocytic activity [32, 33]. Therefore, the phagocytic function of macrophages in tumor is related to the progression of tumor to a certain extent. In our study, contrast agent retention in tumor at KP was found to be negatively correlated with MVI, consistent with above researches. The CEUS feature of KP agent clearance showed a relatively balanced sensitivity (68.3–91.7%) and specificity (70.7–85.2%). This unique feature was first reported in our study and presented great potential as an indicator for MVI prediction.

Previous studies have established several models based on imaging features or biochemical indicators for preoperative assessment of MVI, reaching AUCs of 0.60–0.82 [34–36]. The combined model in our study was constructed based on the combination of multimodal US imaging features and biochemical indicator PIVKA-II, which showed good performance with AUCs of 0.879–0.917. Although the addition of KP agent clearance to the BD-US model based on two conventional features brought a significant increase on sensitivity and AUROC values, a slight decrease could also be found in specificity. The incorporation of PIVKA-II level compensated the lack of specificity and further optimized the model performance with a highest AUROC values among the three models. The MVI probability predicted by the combined model was in good agreement with the actual probability. The combined model could provide a reliable guidance for early aggressive treatment of preoperative predicted MVI-positive patients. In further subgroup analyses the AUC values of the combined model were all higher than 0.90, and no significant differences was found in patients with different liver background and tumor sizes, which showed a stable and powerful performance in MVI prediction.

Our study had several limitations. First, the prediction model did not further stratify MVI-positive patients into mild and severe ones. Although the proportion of patients with severe MVI is very small, preoperative detection of these high-risk patients can further be helpful in treatment strategies making. Second, the imaging indicators included in this study are subjective features interpreted by radiologists. If objective indicators such as radiomics features can be further included, the generalization ability of the model will be enhanced.

In conclusion, the prediction model based on the combination of conventional US, Sonazoid-CEUS, and biochemical indicator can satisfactorily predict MVI-positive in patients with a single HCC ≤ 5 cm. It is a promising tool for preoperative MVI prediction, and further external validation is required prior to application in clinical practice.

## Supplementary information

ELECTRONIC SUPPLEMENTARY MATERIAL

## Data Availability

The datasets used or analyzed during the current study are available from the corresponding authors upon reasonable request.

## Abbreviations

AFP: Alpha-fetoprotein
AP: Arterial phase
AUROC: Area under the receiver operating characteristic curve
CEUS: Contrast-enhanced ultrasound
DP: Delayed phase
HCC: Hepatocellular carcinoma
ICC: Interclass correlation coefficient
KP: Kupffer phase
MVI: Microvascular invasion
PIVKA-II: Vitamin K absence-II
PVP: Portal venous phase
ROC: Receiver operating characteristic curve
