# Supplementary material for: Prediction of microvascular invasion in hepatocellular carcinoma with conventional ultrasound, Sonazoid-enhanced ultrasound, and biochemical indicator: a multicenter study
Source: Insights Imaging. 2024 Oct 28;15:261. doi: 10.1186/s13244-024-01743-3 (PMC11519233; doi:10.1186/s13244-024-01743-3)
Supplement: Supplementary file 1 — ELECTRONIC SUPPLEMENTARY MATERIAL [file 13244_2024_1743_MOESM1_ESM.pdf]

Prediction of microvascular invasion in hepatocellular carcinoma with conventional ultrasound, Sonazoid-enhanced ultrasound, and biochemical indicator

ELECTRONIC SUPPLEMENTARY MATERIAL

Table S1. Qualitative multimodal US imaging features of HCC patients and agreement between observers.

	Observer 1	Observer 2	Cohen's Kappa
Tumor boundary			0.891
Clear	113	117	
Obscure	205	201	
Intra-tumoral artery			0.953
Absence	196	201	
Presence	122	117	
AP enhancement pattern			0.896
Homogeneous	204	209	
Heterogeneous	114	109	
Necrosis			0.957
Absence	280	277	
Presence	38	41	
Capsular enhancement			0.900
Absence	257	255	
Presence	61	63	
KP gross morphology			0.955
Single nodular type	263	265	
Focal extra-nodular type	55	53	
KP agent clearance			
Completely clear	101	98	0.905
Partially clear	217	220	

Abbreviations: AP, arterial phase; KP, Kupffer phase.

Table S2. Quantitative multimodal US imaging features of HCC patients and agreement between observers.

	ICC	*p* value
Tumor diameter, cm	0.998	<0.001
Enhancement onset time, s	0.982	<0.001
Time to Peak, s	0.971	<0.001
Wash-out Time, s	0.950	<0.001

Abbreviations: ICC, interclass correlation coefficient.
